# Identification of key targets driving biofilm formation and virtual screening of potential inhibitors in *Pseudomonas aeruginosa*

**DOI:** 10.3389/fmicb.2026.1867561

**Published:** 2026-07-08

**Authors:** Rui Wang, Enpan Mo, Juqi Wen, Geping Chen, Jingyi Mo, Jun Xu, Wenying Chen

**Affiliations:** 1 Department of Pharmacy, The Third Affiliated Hospital, Southern Medical University, Guangzhou, China; 2 Clinical Research Center, Dermatology Hospital of Southern Medical University, Guangzhou, China; 3 College of Pharmacy, Jinan University, Guangzhou, China

**Keywords:** biofilm, bioinformatics analysis, machine learning, molecular docking, *Pseudomonas aeruginosa*, virtual screening

## Abstract

**Background:**

*Pseudomonas aeruginosa*, a member of the “ESKAPE” pathogens, possesses a robust ability to form biofilms—a key factor that contributes to its antibiotic resistance and poses significant challenges for clinical management. Identifying potential therapeutic targets through bioinformatic analysis of genomic data is therefore critical for developing more effective treatment strategies.

**Methods:**

In this study, five gene expression datasets from the Gene Expression Omnibus were integrated to investigate transcriptional differences between planktonic and biofilm-associated *P. aeruginosa* populations. R was used to identify differentially expressed genes (DEGs), followed by weighted gene co-expression network analysis (WGCNA), to determine significant co-expression modules. Overlapping genes between the DEGs and WGCNA-derived modules were subsequently analyzed to screen for robust hub genes using three machine-learning algorithms: Random Forest, Support Vector Machine-Recursive Feature Elimination, and Least Absolute Shrinkage and Selection Operator. Their diagnostic performance and discriminative ability were evaluated using receiver operating characteristic analysis and boxplot visualization. Furthermore, a systematic literature review was conducted to examine the relationship between *P. aeruginosa* biofilm formation and host immune status, enabling a preliminary assessment of associations between the identified biomarkers and immune cell infiltration. Finally, potential inhibitory compounds were screened from an FDA-approved drug library, validated through molecular docking and molecular dynamics simulations, and subjected to RNA-sequencing (RNA-seq) analysis to assess their effects on biofilm-forming *P. aeruginosa.*

**Results:**

By intersecting the key module genes with the DEGs, a total of 25 overlapping genes were identified, among which wspA emerged as a candidate hub gene functionally associated with *P. aeruginosa* biofilm formation. Virtual screening revealed that DL-menthol exhibits a strong and stable binding affinity toward the wspA protein. RNA-seq further confirmed its significant effects on *P. aeruginosa* during active biofilm formation, with wspA representing a major responsive target. Collectively, these findings provide new insights into therapeutic strategies for biofilm-associated infections, highlight the potential clinical utility of wspA inhibitors, and offer a foundation for developing more effective intervention strategies.

## 1 Introduction

*Pseudomonas aeruginosa*, a Gram-negative, rod-shaped, aerobic bacterium with a single polar flagellum, is a major opportunistic pathogen in clinical settings (Pang et al., 2019). It causes both acute and chronic infections in patients with cystic fibrosis, severe burn injuries, chronic obstructive pulmonary disease, immunodeficiency, and ventilator-associated pneumonia, including COVID-19–related cases. These infections are associated with high morbidity and mortality (Jurado-Martin et al., 2021; Riquelme et al., 2020; Sathe et al., 2023). Recent epidemiological studies indicate a sustained increase in antimicrobial resistance, particularly due to the emergence of multidrug-resistant isolates (Antimicrobial, 2022). Nearly 700,000 deaths worldwide each year are attributed to antibiotic-resistant bacterial infections, posing a major challenge to conventional antimicrobial therapies (Botelho et al., 2019).

Biofilm formation is a major contributor to the antimicrobial resistance of *P. aeruginosa*. The biofilm matrix, composed of polysaccharides, proteins, extracellular DNA, and other extracellular polymeric substances, establishes physical and biochemical barriers that limit antibiotic penetration and hinder immune clearance. Within this protected niche, bacterial cells often transition into slow-growing or dormant states, further enhancing their tolerance to environmental stressors and antimicrobial agents (Cendra and Torrents, 2021; Lau et al., 2005; Yan and Bassler, 2019). Consequently, biofilm-associated infections frequently lead to treatment failure and disease recurrence. Elucidating the molecular mechanisms underlying biofilm development and identifying actionable targets within these pathways are therefore critical for the development of novel therapeutic strategies.

In recent years, bioinformatics (Holtstrater et al., 2020) and machine learning (Jiang et al., 2020) have become powerful tools for dissecting complex regulatory networks and identifying key genes (Chowdhury et al., 2026). Given the multifactorial and highly coordinated nature of *P. aeruginosa* biofilm formation, integrative and systematic analytical strategies are required. In this study, we integrated multiple transcriptomic datasets associated with *P. aeruginosa* biofilm formation and applied various machine-learning algorithms to systematically screen for feature genes. Systematic analysis of bacterial gene expression profiles under different environmental conditions enabled the identification of core regulatory genes closely linked to biofilm development. Their associations with biofilm-related phenotypes were preliminarily assessed.

## 2 Methods

### 2.1 Data collection and processing

**Figure 1:**
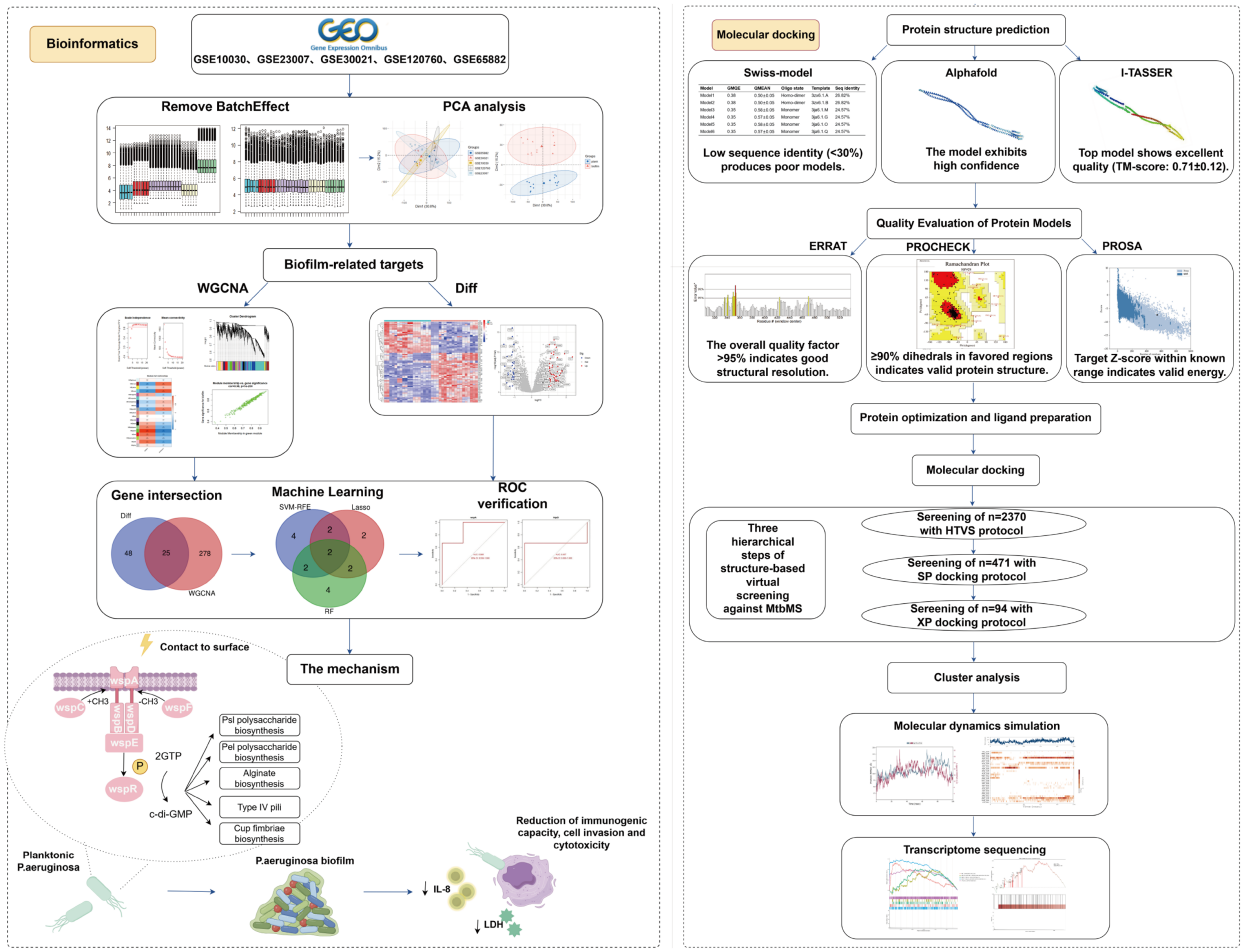
Flowchart depicting the overall study design.

The overall study design is illustrated in Figure 1. Gene expression datasets were retrieved from the Gene Expression Omnibus (GEO, https://www.ncbi.nlm.nih.gov/geo/) by querying the terms “*Pseudomonas aeruginosa*” [porgn], “biofilm” [MeSH Terms], and “Expression profiling by array” [All Fields]. The inclusion criteria were as follows: microarray datasets containing (i) both biofilm and planktonic samples of *P. aeruginosa* samples, (ii) untreated samples, and (iii) ≥2 samples per group.

All datasets meeting these criteria were systematically collected. Datasets involving drug-treated conditions or lacking either biofilm or planktonic groups were excluded. Based on these criteria, six datasets (GSE10030, GSE23007, GSE30021, GSE120760, GSE65882, and GSE12207) were selected. The first five were used as discovery sets, while GSE12207 served as a validation set (Table 1). Additionally, GSE12245, comprising transcriptomic profiles of human alveolar macrophages following *P. aeruginosa* infection, was included to identify immune-related differentially expressed genes (DEGs). All subsequent analyses were performed using R software (version 4.4.1).

**Table 1:** Characteristics of microarray datasets obtained from GEO.

GEO dataset	Platform	Treatment	Control
GSE10030	GPL84	3	2
GSE23007	GPL84	6	6
GSE30021	GPL84	3	3
GSE120760	GPL84	3	3
GSE65882	GPL84	3	4
GSE12207	GPL84	3	3
GSE12245	GPL80-30376	3	3

### 2.2 Batch effect correction and data integration

The GSE10030, GSE23007, GSE30021, GSE120760, and GSE65882 datasets were integrated. Raw expression matrices were first preprocessed using the “limma” package in R, followed by quantile normalization with the normalizeBetweenArrays function to obtain a unified expression matrix. Batch effects were subsequently corrected using the ComBat function from the “sva” package under a parametric empirical Bayes framework with default settings. To assess the effectiveness of batch correction, distribution plots of expression values and principal component analysis were performed before and after correction.

### 2.3 Identification of DEGs

After batch effect correction, samples from multiple GEO datasets were merged into a single integrated expression matrix. Within this corrected combined dataset, samples were categorized into two biological conditions according to their original annotations: the biofilm group and the planktonic group. DEGs between the biofilm and planktonic groups were identified using the “limma” package in R (Ritchie et al., 2015). Multiple testing correction was performed using the Benjamini–Hochberg method, and to minimize false-positive results, adjusted *p*-values were used. Genes with an adjusted *p* < 0.05 and |log₂(fold change)| > 1 were defined as DEGs. A volcano plot and a heatmap were generated in R to visualize the overall distribution and expression patterns of these DEGs.

### 2.4 Functional enrichment analysis

Functional enrichment analysis was performed to characterize the potential biological roles of the identified candidate targets. Gene Ontology (GO) analysis was used to annotate genes with functional attributes across the domains of molecular function (MF), biological process (BP), and cellular component (CC). Further, Kyoto Encyclopedia of Genes and Genomes (KEGG) enrichment analysis was conducted to identify high-level genomic information and signaling pathways related to these functions. All enrichment analyses were performed using the DAVID database based on over-representation analysis (ORA), with a significance threshold of adjusted *p* < 0.05 (Benjamini correction) to ensure the robustness and reliability of functional annotations and pathway interpretations (Huang et al., 2009; Sherman et al., 2022).

### 2.5 Construction of coexpressed gene modules

Weighted gene co-expression network analysis (WGCNA) approach (Langfelder and Horvath, 2008) was used to characterize gene co-expression patterns across different conditions. Sample clustering was first performed to detect outliers. Prior to network construction, soft-thresholding power was selected based on scale-free topology criteria, with R^2^ ≥ 0.85 used to ensure scale-free network properties. The adjacency matrix was transformed into a topological overlap matrix, followed by hierarchical clustering and module detection using the dynamic tree-cutting algorithm. Module eigengenes were correlated with phenotypic traits using Pearson correlation analysis to identify the module most associated with biofilm formation. Genes within this key module were then intersected with differentially expressed genes to obtain candidate targets potentially involved in *P. aeruginosa* biofilm formation.

### 2.6 Screening and validation of diagnostic markers

New potential biomarkers for *P. aeruginosa* biofilm formation were identified using three machine learning algorithms: Random Forest (RF), Least Absolute Shrinkage and Selection Operator (LASSO) logistic regression, and Support Vector Machine-Recursive Feature Elimination (SVM-RFE). RF analysis was performed using the “randomForest” package in R (Liaw and Wiener, 2002), with a random seed set to 12345 to ensure reproducibility. Genes were ranked according to their MeanDecreaseGini values, and the top-ranked features were retained as candidate variables. LASSO logistic regression was conducted with the R package “glmnet” (Engebretsen and Bohlin, 2019) R package, with the optimal lambda value determined by minimizing partial likelihood deviance via 10-fold cross-validation. SVM-RFE was conducted using the “e1071” package, applying recursive feature elimination to select the optimal feature subset. Genes identified by all three algorithms were selected as candidate biomarkers.

The predictive performance of these candidates was evaluated using GSE12207 as an independent validation set. Receiver operating characteristic (ROC) curves and the corresponding area under the curve (AUC) (Tang et al., 2023) were used to assess predictive capability, and statistical significance was set at a two-tailed *p* < 0.05.

### 2.7 Construction of a network for wspA-mediated biofilm formation and its potential regulation of immune-related genes

WspA-associated proteins were obtained from STRING (Szklarczyk et al., 2023) and corresponding genes were subjected to KEGG enrichment analysis using DAVID (adjusted *p* < 0.05, Benjamini correction). In parallel, differentially expressed genes (DEGs) from human immune-related microarray datasets were analyzed using the same pipeline. To investigate potential links between wspA and host immunity, literature-curated interactions between immune-related genes and biofilm formation were integrated. A protein–protein interaction (PPI) network was constructed by combining wspA-associated genes and immune DEGs, with biofilm formation as a functional intermediary to assess indirect associations.

### 2.8 Homology modeling

The wspA protein sequence was retrieved from the NCBI database in FASTA format. Three-dimensional structures were predicted using Swiss-Model,^1^ I-TASSER,^2^ and AlphaFold,^3^ Stereochemical quality, model accuracy, and energy rationality were evaluated using PROCHECK, ERRAT, and ProSA, respectively, via SAVES.^4^ The model meeting acceptable quality based on these assessments was selected as the final structure.

### 2.9 Molecular docking

Structure-guided virtual screening was performed to identify potential anti-wspA compounds, providing an efficient and cost-effective alternative to experimental high-throughput screening. The workflow was implemented using the Glide module in Schrödinger (Halgren et al., 2004), and included three successive stages: high-throughput virtual screening (HTVS), standard precision (SP), and extra precision (XP) docking. In the HTVS stage, the top 20% of compounds were retained to rapidly eliminate unfavorable conformations and reduce the computational burden. The selected subset was then subjected to SP docking to remove false-positive binders and further refine ligand prioritization. Finally, the top-ranked compounds were docked using the XP protocol, which applies stringent scoring and conformational sampling to optimize binding poses and reduce misleading matches (Friesner et al., 2006). The best complexes were selected based on Glide scores, and their binding modes were visualized for subsequent experimental validation.

### 2.10 Molecular dynamics simulation analysis

Molecular dynamics simulations were performed using the Schrödinger Desmond software (Schrödinger, 2023). The protein–ligand complex was placed in a cubic water box constructed using the SPC water model. Sodium and chloride ions were added to neutralize the system, with an additional salt concentration of 0.15 M to mimic physiological ionic conditions. Energy minimization was conducted using the OPLS4 force field to ensure physically reasonable initial conformations (Rivera-Sanchez et al., 2022). The system was then gradually heated and equilibrated at 300 K and 1 atm. After complete relaxation, the system was subjected to a 100 ns molecular dynamics simulation. The binding free energy of the complex under steady-state conditions was calculated using the Schrödinger thermal_mmgbsa.py script. All other simulation parameters were maintained at their default values.

### 2.11 Biofilm quantification by crystal violet (CV) assay

Overnight cultures of *Pseudomonas aeruginosa* PAO1 were diluted in Luria–Bertani (LB) medium to an OD600 of 0.02. Menthol was dissolved in Ammonium–Basal (AB) medium using dimethyl sulfoxide (DMSO) as a solvent, with the final concentration of DMSO in all experimental conditions not exceeding 0.1% (v/v). Then, 100 μL of bacterial suspension and 100 μL of either menthol-containing solution or control solution were added to 96-well plates. The experimental design included an untreated bacterial control group and a vehicle control containing the corresponding concentration of DMSO without menthol. Plates were incubated at 37 °C under static conditions for 24 h to allow biofilm formation.

After incubation, wells were washed three times with PBS, air-dried, and fixed with methanol for 15 min. Biofilms were stained with 0.1% crystal violet for 15 min, washed, and completely dried. The bound dye was solubilized in 30% acetic acid, and absorbance was measured at 570 nm. All experiments were performed in triplicate. Statistical analysis was conducted using one-way analysis of variance (one-way ANOVA), followed by Dunnett’s multiple-comparison test to compare each treatment group with the vehicle control group. Differences were considered statistically significant at *p* ≤ 0.05.

### 2.12 Transcriptome sequencing

*Pseudomonas aeruginosa* PAO1 was cultured in 5 mL of LB medium and incubated overnight at 37 °C. The following day, 1 mL of the overnight culture was inoculated into 100 mL of fresh AB medium at a 1:100 dilution and grown to logarithmic phase.

For transcriptomic analysis, bacterial cultures were divided into treatment and vehicle control groups. For the treatment group, 100 μL of the compound solution was added. For the control group, an equal volume of solvent (vehicle) without the compound was added to ensure identical solvent exposure across conditions. All experimental groups were prepared in biological triplicate.

Cultures were further incubated at 37 °C with shaking at 200 rpm for 5 h. Cells were harvested by centrifugation at 4 °C at 5,000 rpm for 5 min. Cell pellets were immediately flash-frozen in liquid nitrogen and stored at −80 °C until further processing. Total RNA extraction, library preparation, and transcriptome sequencing were performed by Novogene Co., Ltd. (Beijing, China) using the Illumina X Plus platform following standard procedures.

## 3 Results

### 3.1 DEG screening and data preprocessing

**Figure 2:**
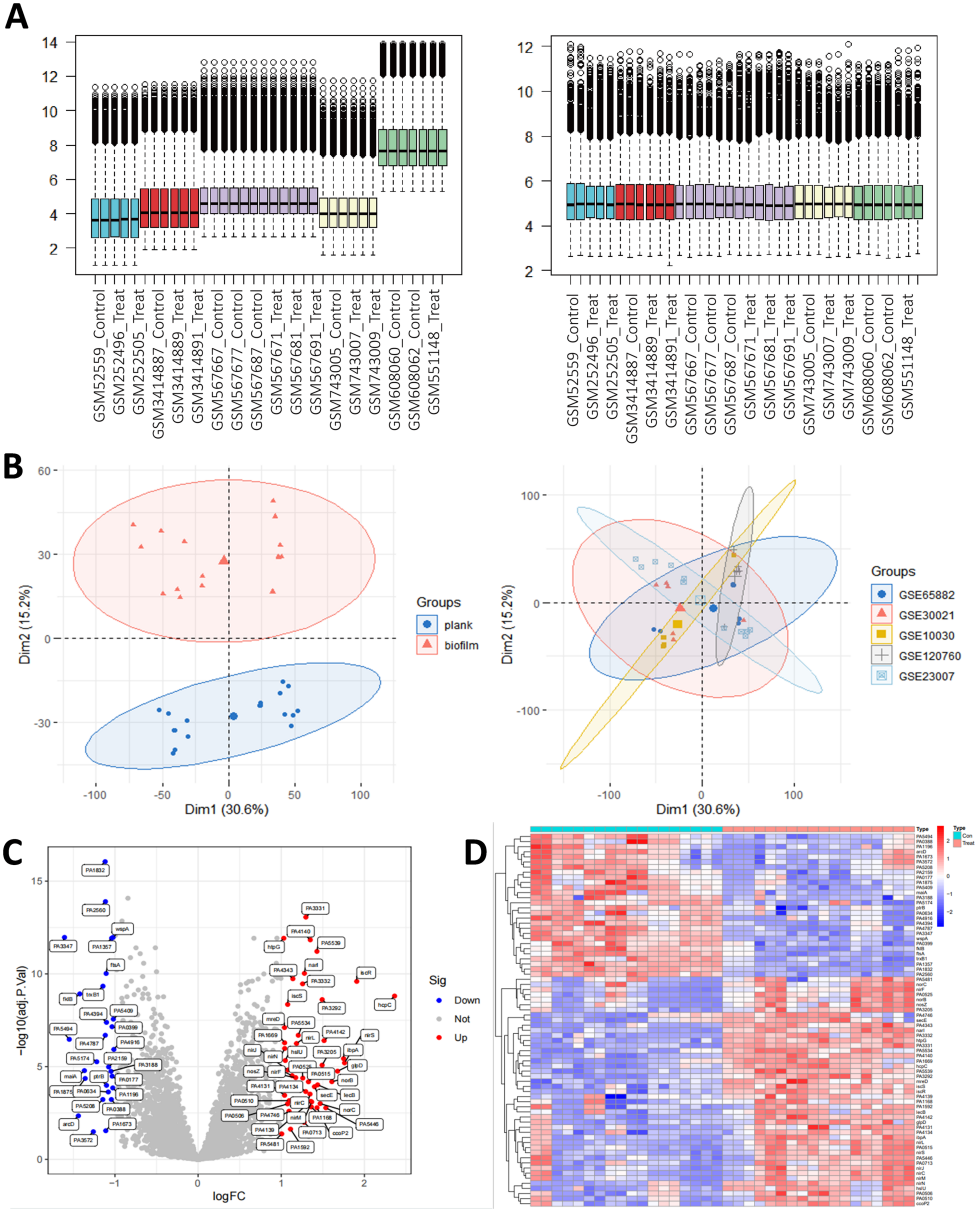
Data preprocessing and identification of differentially expressed genes. **(A)** Box plots of normalized gene expression values across samples. **(B)** Principal component analysis of all samples. **(C)** Volcano plot showing upregulated and downregulated genes. **(D)** Heatmap showing expression patterns of differentially expressed genes.

Box plots of standardized expression data, with different colors representing distinct data sets, illustrate the distribution of *P. aeruginosa* biofilm microarray data before and after normalization and batch effect correction (Figure 2A). Principal component analysis showed clear separation between biofilm and planktonic samples, indicating pronounced differences in overall gene expression. Samples from different genomic backgrounds partially overlapped, suggesting sufficient comparability for integrative analysis (Figure 2B). Under the criteria of *p* < 0.05 and |log₂ fold-change| > 1, a total of 73 DEGs were identified, including 45 up-regulated and 28 down-regulated genes. Their distribution and relative expression patterns are illustrated in the volcano plot and heatmap (Figures 2C, D).

### 3.2 Functional enrichment analysis of DEGs

**Figure 3:**
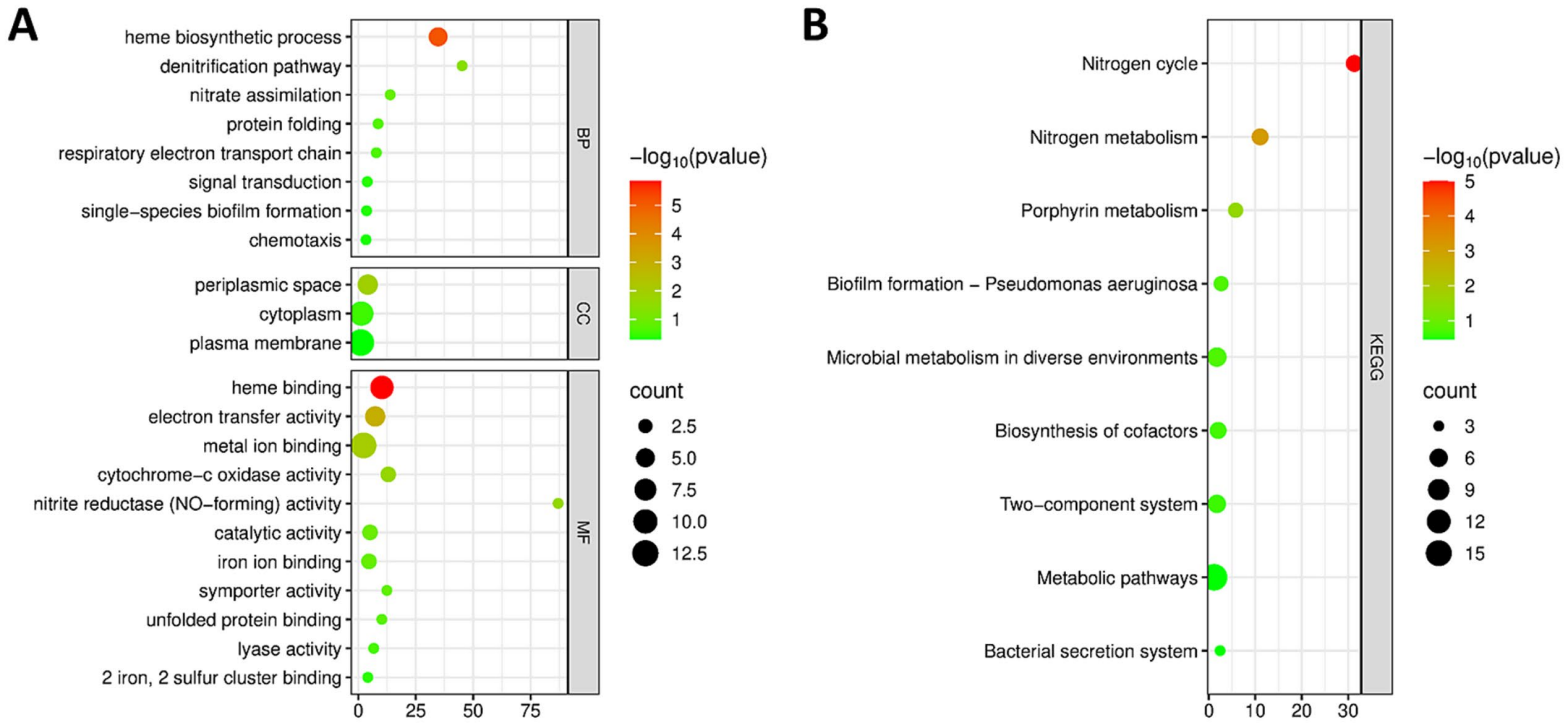
Functional enrichment analysis of DEGs. **(A)** Gene Ontology (GO) and **(B)** Kyoto Encyclopedia of Genes and Genomes (KEGG) pathway enrichment analyses.

To predict the biological functions of the DEGs, we performed functional enrichment analysis (Figure 3A). The BP terms were primarily enriched in heme biosynthesis, denitrification and nitrate assimilation pathways, respiratory electron transport, signal transduction, and processes directly involved in single-species biofilm formation and chemotaxis.

MF enrichment included electron transfer activity, heme and metal ion binding, cytochrome *c* oxidase activity, and nitrite reductase activity. CC terms were primarily associated with the periplasmic space, cytoplasm, and plasma membrane. KEGG pathway analysis revealed enrichment in nitrogen metabolism, porphyrin metabolism, two-component systems, bacterial secretion systems, and the *P. aeruginosa* biofilm formation pathway (Figure 3B).

### 3.3 WGCNA implementation and identification of key module genes

**Figure 4:**
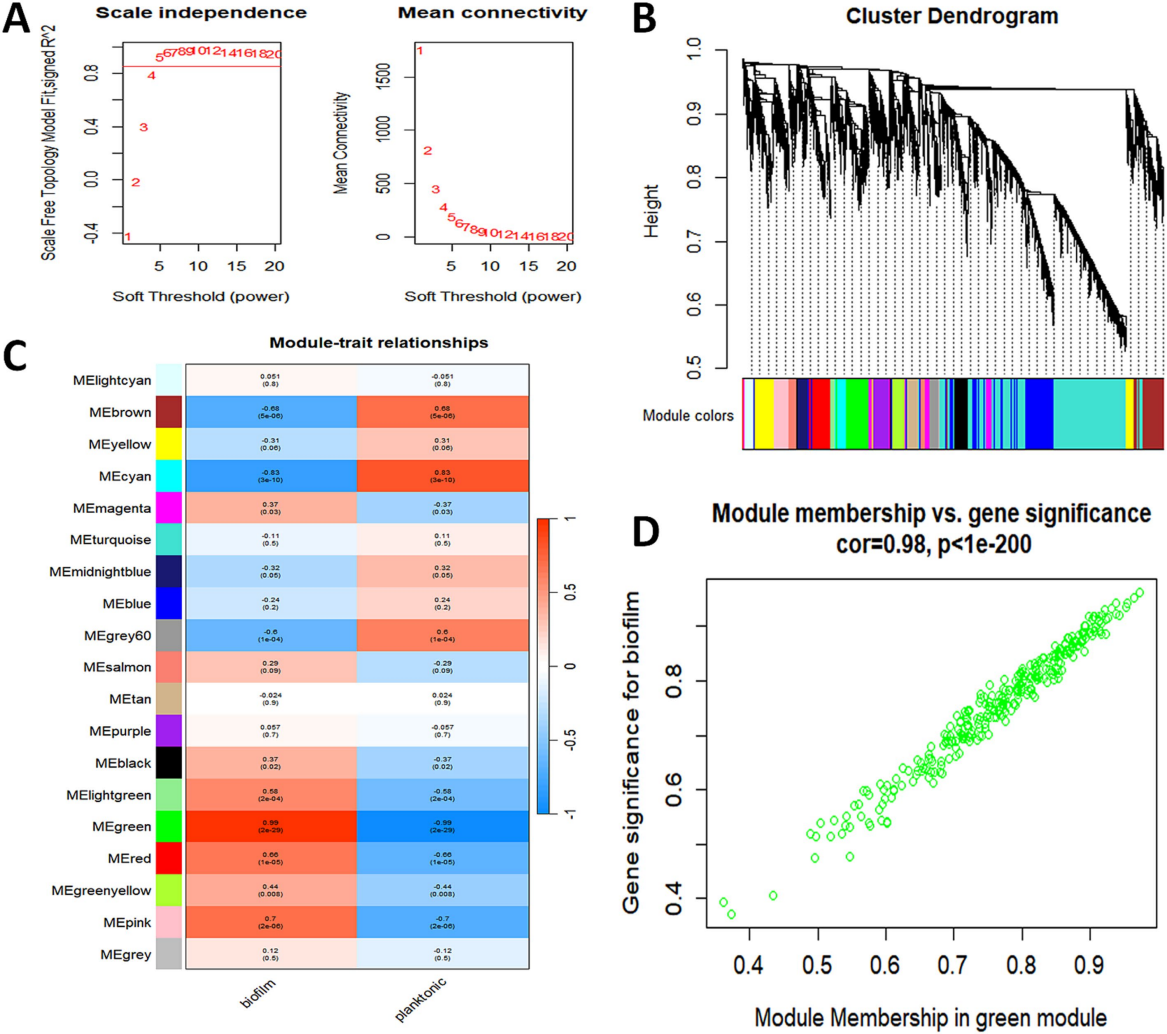
Construction of the weighted gene co-expression network associated with biofilm formation in *Pseudomonas aeruginosa*. **(A)** Selection of soft-thresholding power (*β* = 5) based on scale-free topology. **(B)** Hierarchical clustering dendrogram of module eigengenes. **(C)** Heatmap of module–trait correlations. **(D)** Scatterplot for the green module showing the relationship between module membership and gene significance.

To identify gene networks associated with biofilm formation, a weighted gene co-expression network analysis (WGCNA) was performed using *Pseudomonas aeruginosa* transcriptomic datasets related to biofilm growth conditions. Samples from five public datasets were categorized into biofilm and planktonic groups to enable phenotype-associated network analysis. Based on the scale-free topology criterion (*R*^2^ > 0.85), a soft-thresholding power of *β* = 5 was selected to construct a scale-free co-expression network (Figure 4A).

Subsequently, hierarchical clustering of genes combined with the dynamic tree-cutting algorithm identified a total of 19 distinct co-expression modules, each representing a group of genes with highly correlated expression patterns (Figures 4B, C). These modules were further analyzed to determine their association with the biofilm phenotype.

Module–trait correlation analysis revealed that the green module exhibited the strongest positive correlation with biofilm formation (correlation coefficient = 0.98, *p* < 0.05) (Figure 4D). This module contained 303 genes, suggesting that these genes may participate in coordinated regulatory processes involved in biofilm development.

### 3.4 DEGs and functional analysis of critical module genes

**Figure 5:**
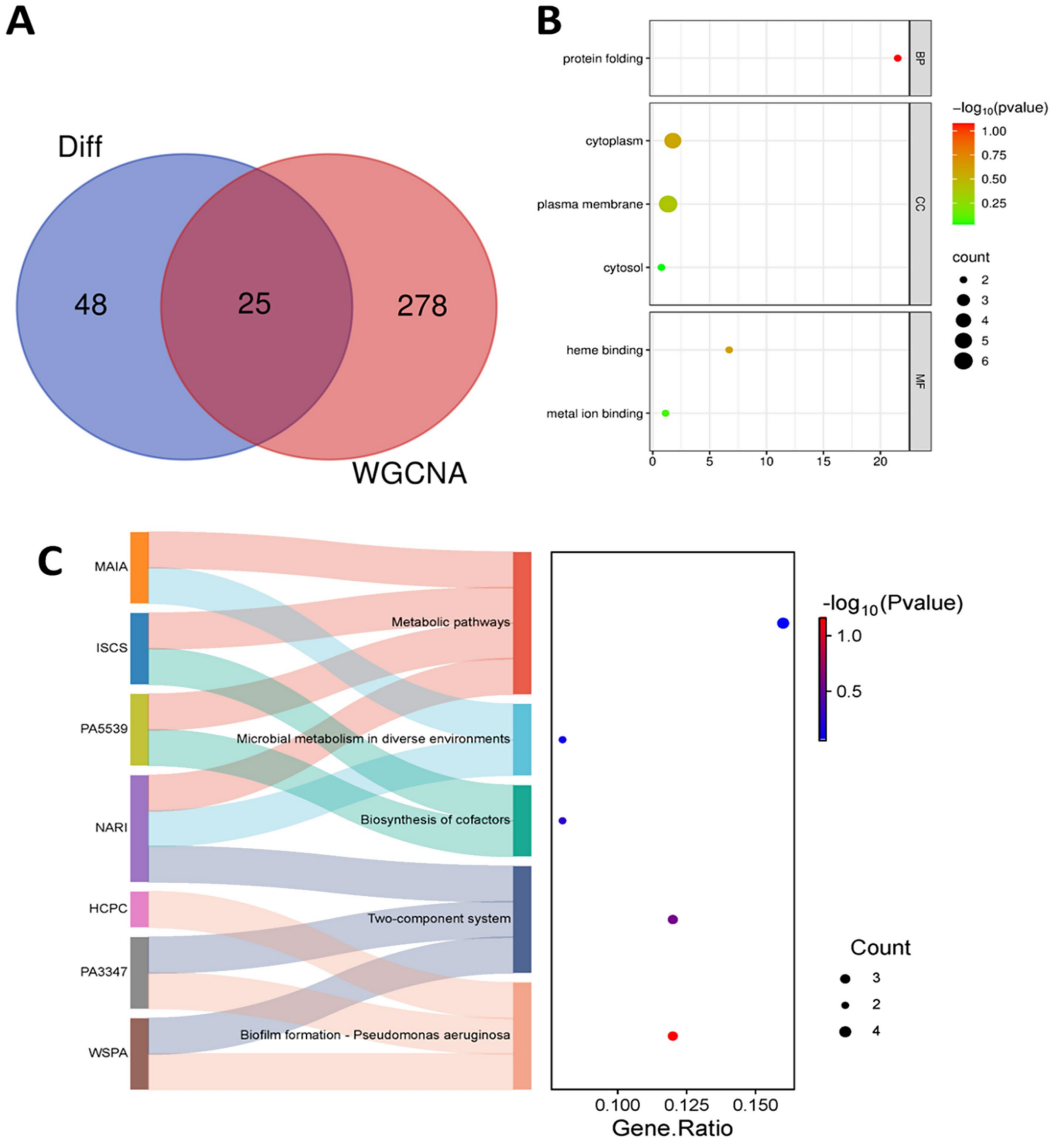
Functional analysis of key genes overlapping with differentially expressed genes. **(A)** Venn diagram showing overlap between key module genes and differentially expressed genes. **(B)** Gene Ontology enrichment analysis in biological process, cellular component, and molecular function categories. **(C)** Kyoto Encyclopedia of Genes and Genomes pathway enrichment analysis.

Overlap analysis between critical module genes and DEGs identified 25 shared genes, as shown in the Venn diagram (Figure 5A). We examined the biological roles of these overlapping genes through functional enrichment analysis.

Gene Ontology (GO) enrichment analysis showed that these genes were significantly enriched in molecular functions related to heme binding and electron transfer activity. Although other categories, including cytoplasm, plasma membrane, and metal ion binding, did not reach statistical significance, they may still reflect potential functional trends of the overlapping genes (Figure 5B).

KEGG pathway analysis revealed significant enrichment in pathways associated with the nitrogen cycle and nitrogen metabolism. Meanwhile, several additional pathways, including *Pseudomonas aeruginosa* biofilm formation, the two-component system, and microbial metabolism in diverse environments, were also identified but did not reach statistical significance; nevertheless, they were retained to illustrate potential biological relevance and overall functional trends (Figure 5C).

### 3.5 Screening and validation of diagnostic markers

**Figure 6:**
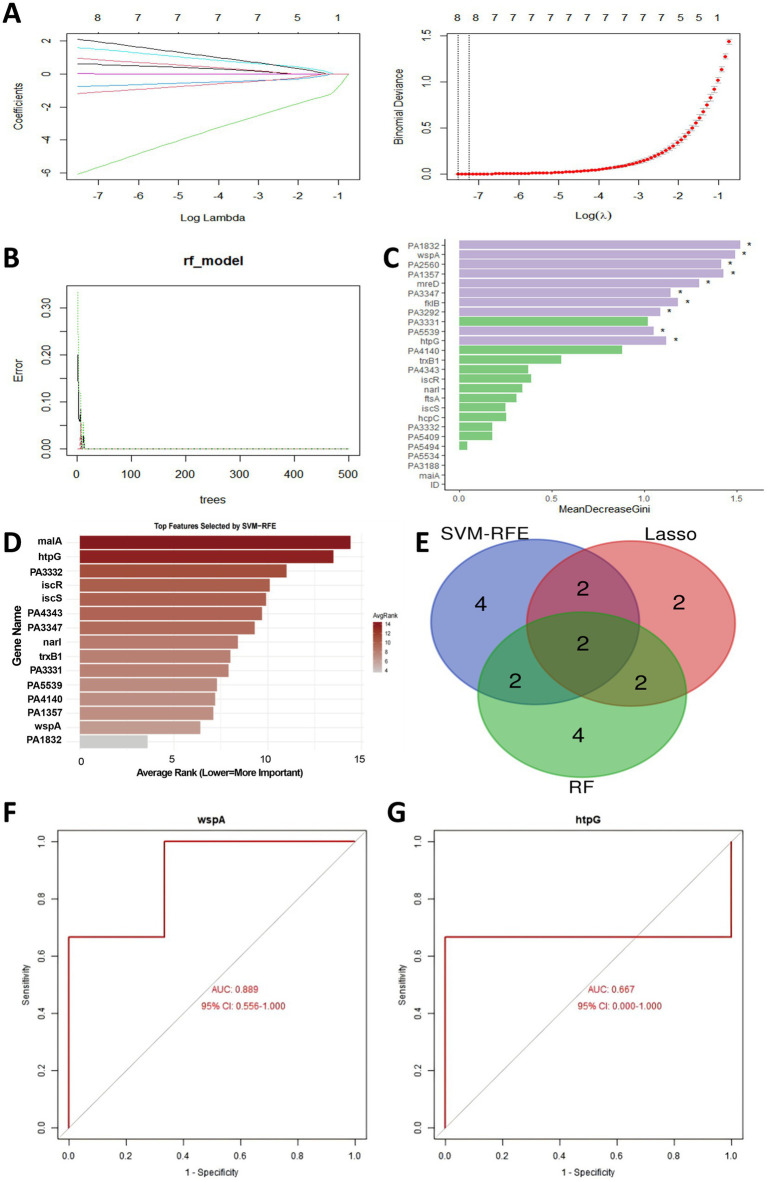
Selection and validation of feature genes. **(A)** Tuning of feature selection in the Least Absolute Shrinkage and Selection Operator model. **(B)** Random Forest error rate as a function of the number of classification trees. **(C)** Top 10 statistically significant genes ranked by relative importance. **(D)** Screening of biomarkers using Support Vector Machine Recursive Feature Elimination. **(E)** Venn diagram showing overlap of genes identified by all three algorithms. **(F,G)** Receiver operating characteristic curves validating the genes wspA **(F)** and htpG **(G)**.

We applied three machine learning algorithms to identify candidate feature genes. LASSO regression analysis selected eight predicted genes from statistically significant univariate variables (Figure 6A). Random Forest analysis combined with feature selection ranked genes based on error rate, number of classification trees, and relative importance, with 10 genes reaching statistical significance (Figures 6B, C). Additionally, SVM-RFE was used to select the top 10 optimal genes through recursive feature elimination (Figure 6D).

A Venn diagram revealed two overlapping genes identified by all three approaches (Figure 6E). In the independent validation dataset GSE12207, ROC analysis showed that wspA and htpG achieved AUC values of 0.889 and 0.667, respectively (Figures 6F, G). Although both genes showed consistent performance across models, wspA exhibited relatively stronger discriminative ability in the validation cohort. Accordingly, wspA was prioritized as the key hub gene, while htpG was retained as a potential candidate requiring further validation.

### 3.6 Comprehensive functional and immune-related network analysis of key regulatory genes

**Figure 7:**
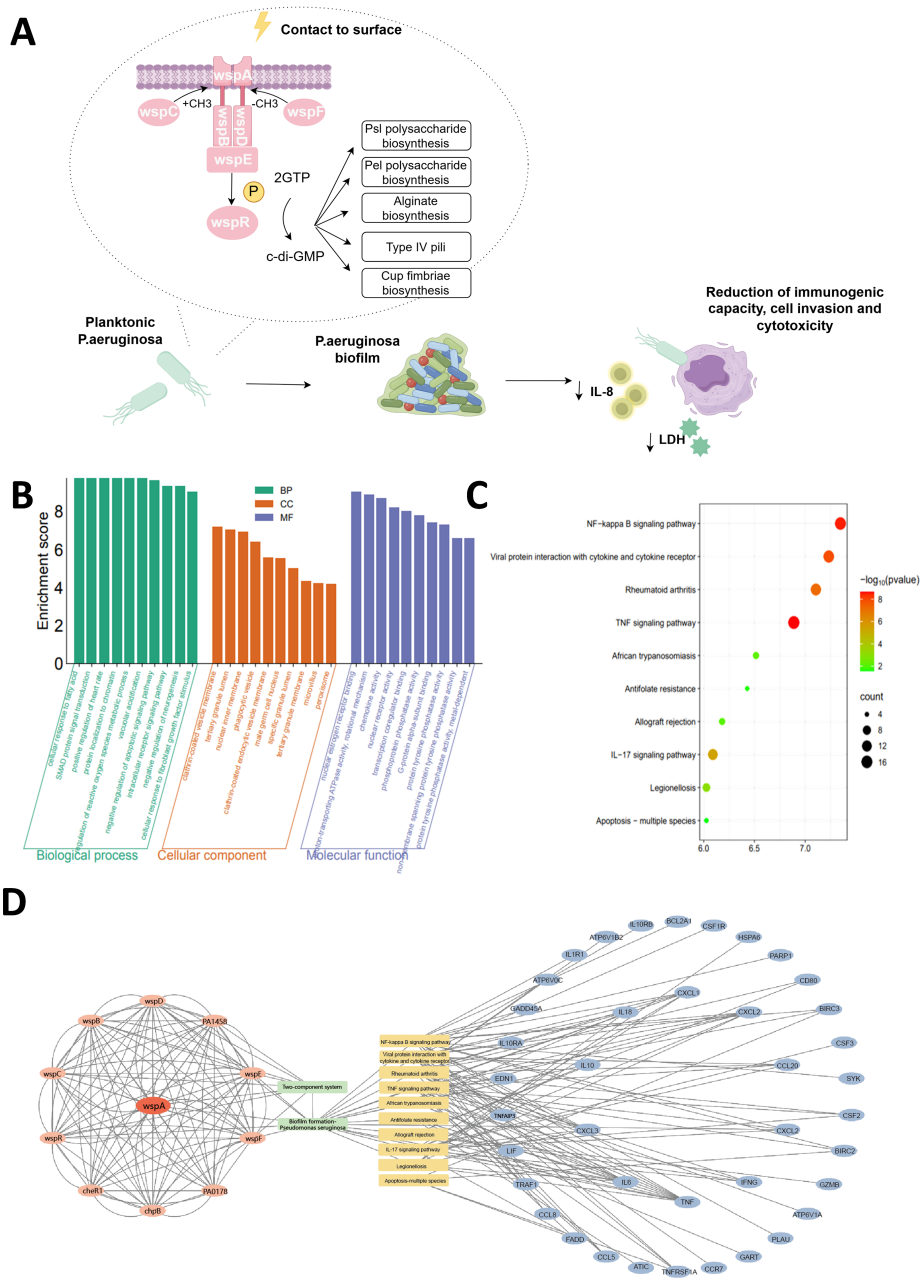
Functional role of wspA and immune-related protein–protein interaction (PPI) network analysis. **(A)** Functional analysis of wspA. **(B)** Gene Ontology enrichment bar plot of immune-related differentially expressed genes. **(C)** Kyoto Encyclopedia of Genes and Genomes enrichment bubble plot of immune-related differentially expressed genes. **(D)** PPI network linking wspA, biofilm formation, and immune-related genes.

As illustrated in Figure 7A, upon suface contact, the membrane-bound receptor wspA activates downstream signaling, leading to WspR activation and elevated intracellular c-di-GMP levels (Güvener and Harwood, 2007; Huangyutitham et al., 2013; O’Connor et al., 2012). The increased c-di-GMP subsequently promotes the production of exopolysaccharides, adhesins, pili, and other matrix components, which together establish a stable and mature biofilm (Baraquet and Harwood, 2013; Irie et al., 2012; Kuchma et al., 2012). This biofilm enhances bacterial resistance to host defenses by forming physical barriers, limiting cell detachment, increasing antimicrobial tolerance, and shielding surface antigens.

Protein–protein interaction analysis indicated that wspA is closely associated with biofilm formation and the two-component regulatory system in *Pseudomonas aeruginosa* (Figure 7D). Analysis of the human immune-related microarray dataset GSE12245 (including three uninfected controls and three *P. aeruginosa*-infected alveolar macrophage samples) showed significant activation of the NF-κB, IL-17, and TNF signaling pathways (Figures 7B,C). Previous studies have reported that biofilm-forming *P. aeruginosa* can modulate host immune responses and induce the expression of pro-inflammatory cytokines, including IL-1β, TNF-α, and IL-6 (Ciornei). WspA may therefore indirectly influence host immune responses by promoting biofilm formation.

### 3.7 Homology modeling

Using the Swiss-Model server (Schwede et al., 2003), 50 potential templates were initially identified, from which six homology models were generated. Although template selection typically requires a sequence identity of ≥ 30%, all six models exhibited sequence identities below this threshold (Table 2). Correspondingly, their (0.35–0.38) and QMEAN (0.50–0.58) scores (Benkert et al., 2011) were relatively low, indicating weak similarity between the target protein and available templates and, consequently, limited overall model reliability.

**Table 2:** Predicted structural models generated by Swiss-Model.

Model	GMQE	QMEAN	Oligo state	Template	Seq identity
Model 1	0.38	0.50 ± 0.05	Homo-dimer	3zx6.1.A	26.82%
Model 2	0.38	0.50 ± 0.05	Homo-dimer	3zx6.1.B	26.82%
Model 3	0.35	0.58 ± 0.05	Monomer	3ja6.1.M	24.57%
Model 4	0.35	0.57 ± 0.05	Monomer	3ja6.1.G	24.57%
Model 5	0.35	0.58 ± 0.05	Monomer	3ja6.1.O	24.57%
Model 6	0.35	0.57 ± 0.05	Monomer	3ja6.1.Q	24.57%

**Figure 8:**
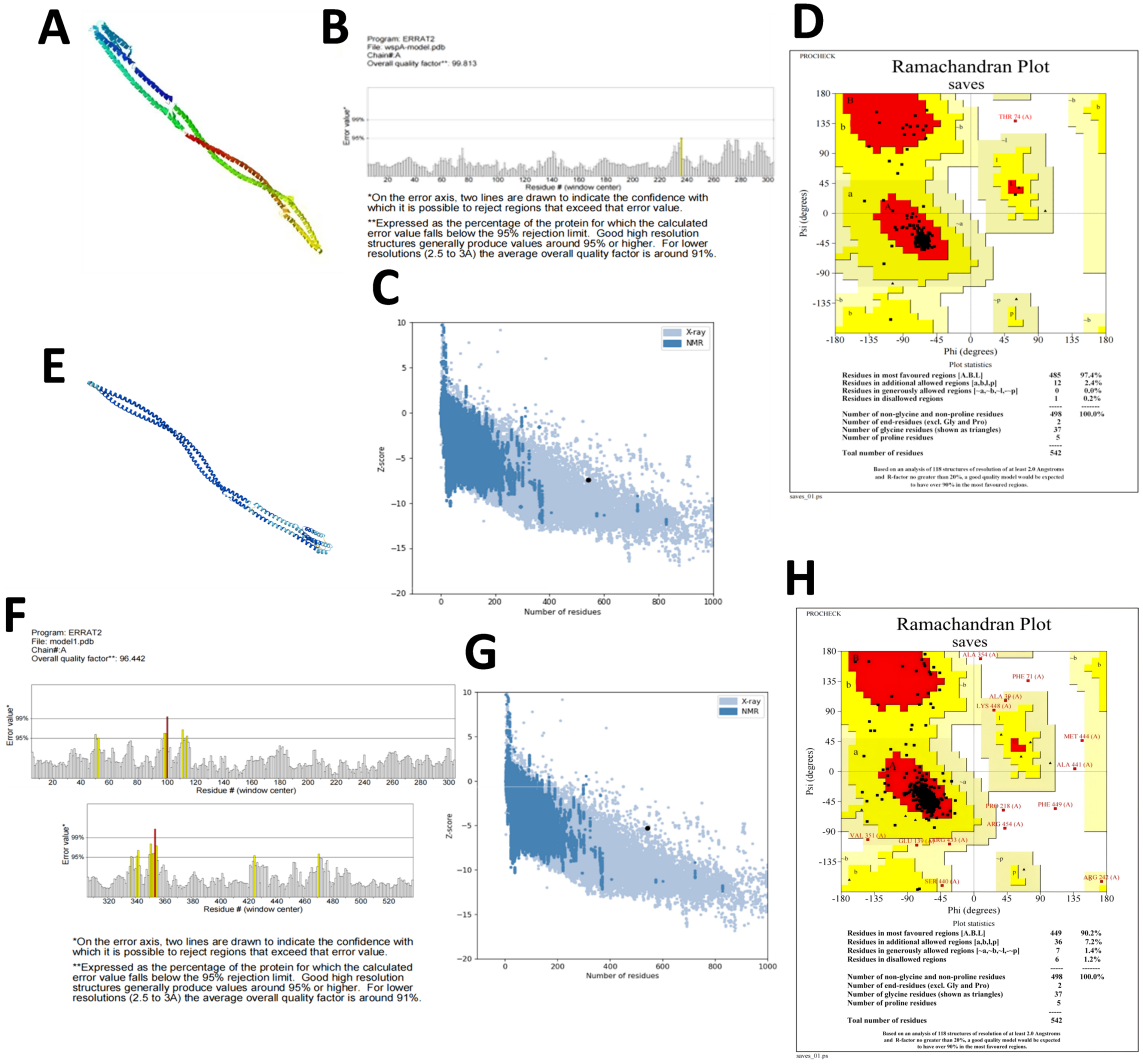
Structural prediction and quality assessment of wspA protein using I-TASSER **(A–D)** and AlphaFold **(E–H)**, including predicted structure **(A,F)**, ERRAT **(B,F)**, ProSA Z-score **(C,G)**, and Ramachandran plot **(D,H)**.

The top three wspA structural models were subsequently generated using I-TASSER (Yang and Zhang, 2015) and ranked based on the proprietary confidence score (C-Score), with values of −0.02, −0.09, and −0.20, respectively. A TM-Score (Zhang and Skolnick, 2004) of 0.71 ± 0.12 was reported only for the highest-ranked model. Because the correlation between C-Score and TM-Score is weak for lower-ranked models, additional correlation metrics were not calculated. Based on overall quality parameters—including C-Score (−0.02), TM-Score (0.71), and cluster size—the highest-ranked I-TASSER model (Model 1) was selected for subsequent structural evaluation and validation (Figure 8A).

To further improve structural prediction, AlphaFold (Cheng et al., 2025) was used to independently model wspA from *P. aeruginosa* (Figure 8E). The resulting structure exhibited an average pLDDT of 90.30, indicating high confidence in the overall fold, with particularly stable regions observed in the transmembrane and signal-transduction-associated domains. These predicted structures were subjected to additional quality assessment.

Structural validation was performed to assess stereochemical quality and suitability for subsequent computational analyses. Model 1 and the AlphaFold-predicted structure were evaluated using PROCHECK (Laskowski et al., 1993), ERRAT (Colovos and Yeates, 1993), and PROSA (Wiederstein and Sippl, 2007). According to PROCHECK, 449 residues (90.2%) in Model 1 and 485 residues (97.4%) in the AlphaFold model fell within the most favored regions, with <1.0% of residues located in disallowed regions in the AlphaFold model.

ERRAT analysis revealed overall quality factors of 96.44% and 99.81% for the I-TASSER and AlphaFold models, respectively—both exceeding the commonly accepted threshold of 50%, with the AlphaFold model achieving the highest score. ProSA results further demonstrated that the Z-scores of the I-TASSER (−5.28) and AlphaFold (−7.38) structures fell within the range of experimentally determined protein structures, confirming reasonable energy profiles.

Collectively, while the I-TASSER model exhibited acceptable reliability, the integrated results from PROCHECK, ERRAT, and ProSA (Figures 8B–D,F) indicate that the AlphaFold-predicted wspA structure shows superior geometric consistency and overall quality. This makes the structure a more robust foundation for downstream docking and molecular dynamics analyses.

### 3.8 Virtual screening

To identify potential wspA inhibitors, key residues (Val223, Leu226, Asp227, Leu240, and Ala230) within the predicted binding pocket were selected as the docking core based on previously reported structural analyses (Skariyachan et al., 2021). A total of 1,810 FDA-approved drugs were subjected to structure-based virtual screening. Initial screening was performed using HTVS to rapidly filter ligands. Compounds passing this stage were further evaluated using SP docking, yielding 471 candidates, of which 94 were selected for XP docking. XP docking provides more thorough refinement and reduces false-positive binding. Finally, the top 20% of compounds ranked by XP docking scores were selected for subsequent analyses.

To identify representative candidate compounds, clustering analysis was performed on the XP docking outputs, yielding four clusters. Previous studies have shown that trimethylangelicin derivatives significantly attenuate inflammation in acute infection models of *P. aeruginosa* without evident toxicity, whereas menthol exerts antibacterial activity primarily through membrane disruption. Based on this evidence, trioxsalen and menthol were selected as representative compounds from Cluster 4 (Table 3). In addition, considering our preliminary findings indicating limited efficacy of desmopressin acetate, DL-menthol (DL-MT), trioxsalen (TMP), colistin sulfate (CMS), and ceftaroline fosamil (CPT-F) were ultimately chosen for further investigation.

**Table 3:** Clustering results of the top 20% ranked compounds.

Cluster	Drug Candidates	CAS	Docking score
Cluster 1	Colistin sulfate	1264-72-8	−5.00763
Cluster 2	Ceftaroline fosamil	400827-46-5	−4.51419
Cluster 3	Desmopressin acetate	62288-83-9	−4.47561
Cluster 4	Trioxsalen	3902-71-4	−4.30651
	Haloperidol	52-86-8	−4.25291
	Stiripentol	49763-96-4	−4.18683
	Revefenacin	864750-70-9	−4.15775
	Oxcarbazepine	28721-07-5	−4.11147
	DL-Menthol	89-78-1	−4.06068
	Rucaparib monocamsylate	1859053-21-6	−4.04773
	Rucaparib Phosphate	459868-92-9	−4.04773
	Estradiol	50-28-2	−3.86932
	Quinidine sulfate dihydrate	6591-63-5	−3.86669

### 3.9 Molecular dynamics simulations

**Figure 9:**
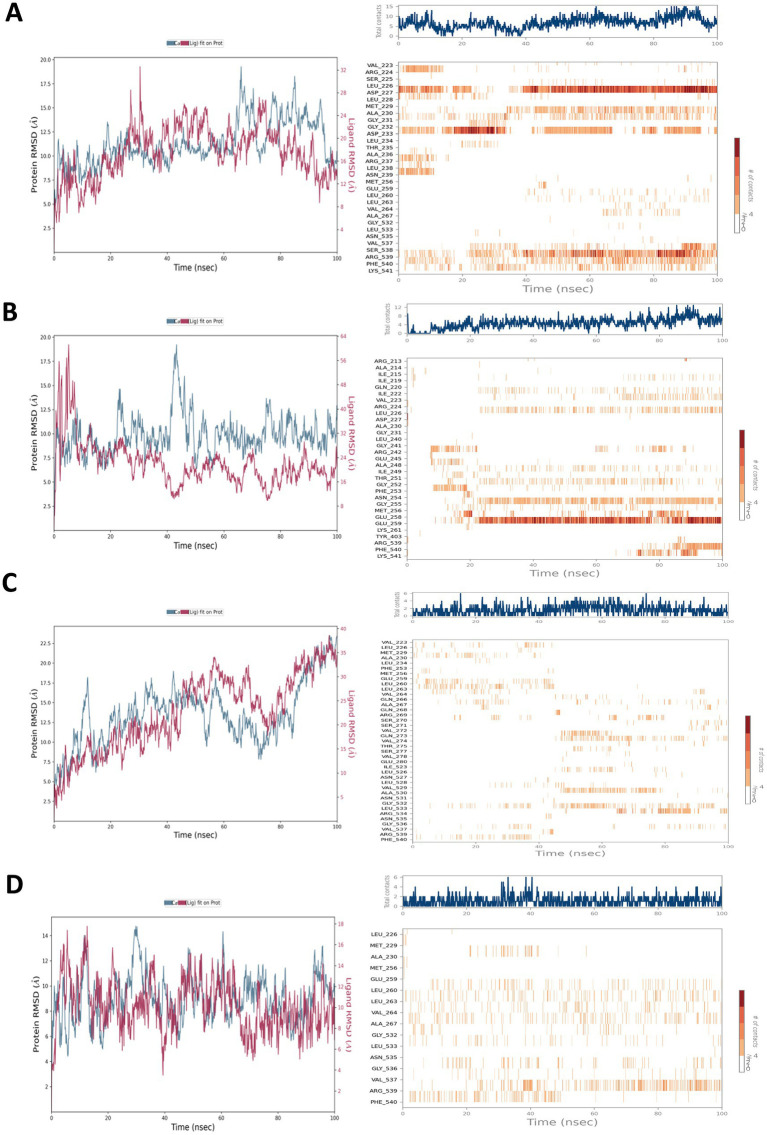
RMSD of protein backbone and ligands (blue: backbone, red: ligand) and ligand–protein interaction analysis for **(A)** CMS, **(B)** CPT-F, **(C)** TMP, and **(D)** DL-MT.

To further evaluate the stability of candidate compounds bound to wspA, molecular dynamics simulations were performed. Most protein–ligand complexes gradually reached equilibrium at approximately 80 ns. Given that wspA is a large, multi-domain methyl-accepting chemotaxis–like receptor (~500 amino acids), the root mean square deviation (RMSD) values of both protein and ligand were ultimately maintained within 4 Å, indicating overall binding stability between wspA and the candidate compounds (Figures 9).

During the simulation, TMP exhibited significant instability when bound to wspA, as reflected by large RMSD fluctuations of both the protein and ligand, which prevented the maintenance of a stable binding mode. Consequently, TMP was excluded from subsequent binding free energy calculations. In contrast, the other three candidate compounds demonstrated comparatively higher stability and were therefore retained for MM/GBSA analysis.

Specifically, CMS formed hydrogen bonds, ionic interactions, and water-bridged interactions with ASP227 and ASP233. ALA230 further contributed through hydrogen bonds, water bridges, and hydrophobic interactions, all of which were crucial for stabilizing the CMS–wspA complex. After 40 ns, PHE539 established additional hydrogen bonds, ionic interactions, and water bridges with CMS, while ARG540 further reinforced complex stability through hydrogen bonding and hydrophobic interactions.

CPT-F gradually interacted with GLU259, GLY255, and GLY252 through hydrogen bonds and water bridges at approximately 20 ns. GLU259 and GLY255 also participated in ionic interactions. As the simulation progressed, GLU258 formed hydrogen bonds, ionic interactions, and water bridges after 75 ns, while PHE540 mainly contributed hydrophobic interactions, further stabilizing the complex.

DL-MT showed a rapid trend toward RMSD stabilization, although the formation of key interactions lagged slightly. Initially, DL-MT formed hydrogen bonds and water-bridged interactions with ARG539 and GLY536, as well as hydrogen and hydrophobic bonds with LEU263. Over time, additional hydrophobic interactions formed with LEU260, VAL264, ALA267, and PHE540, resulting in a stable binding conformation.

Binding free energies were calculated for the three candidate compounds once stable conformations with wspA were reached. GMS (−40.07 kcal/mol), CPT-F (−59.01 kcal/mol), and DL-MT (−28.83 kcal/mol) all exhibited favorable binding free energies, further supporting the stability of their binding modes.

### 3.10 Transcriptomic analysis

In this study, we aimed to evaluate the regulatory effects of the screened candidate compounds on *Pseudomonas aeruginosa* biofilm formation. CMS and CPT-F, as established antibiotics, may exert their observed biofilm inhibitory effects partly through non-specific antibacterial pressure. Accordingly, DL-menthol, which exhibits relatively weak antibacterial activity, was selected as a representative compound to investigate its global transcriptional response and underlying mechanisms using RNA-seq transcriptomic analysis.

To determine the appropriate treatment concentration, crystal violet staining was used to assess biofilm formation under different doses of DL-menthol. The results showed that biofilm biomass remained at a relatively stable inhibitory level when the concentration was below 1,250 μg/mL. Based on these findings, 39 μg/mL was selected as the working concentration for subsequent experiments. This concentration is well below the minimum inhibitory concentration, yet it produced a noticeable inhibitory effect on biofilm formation.

**Figure 10:**
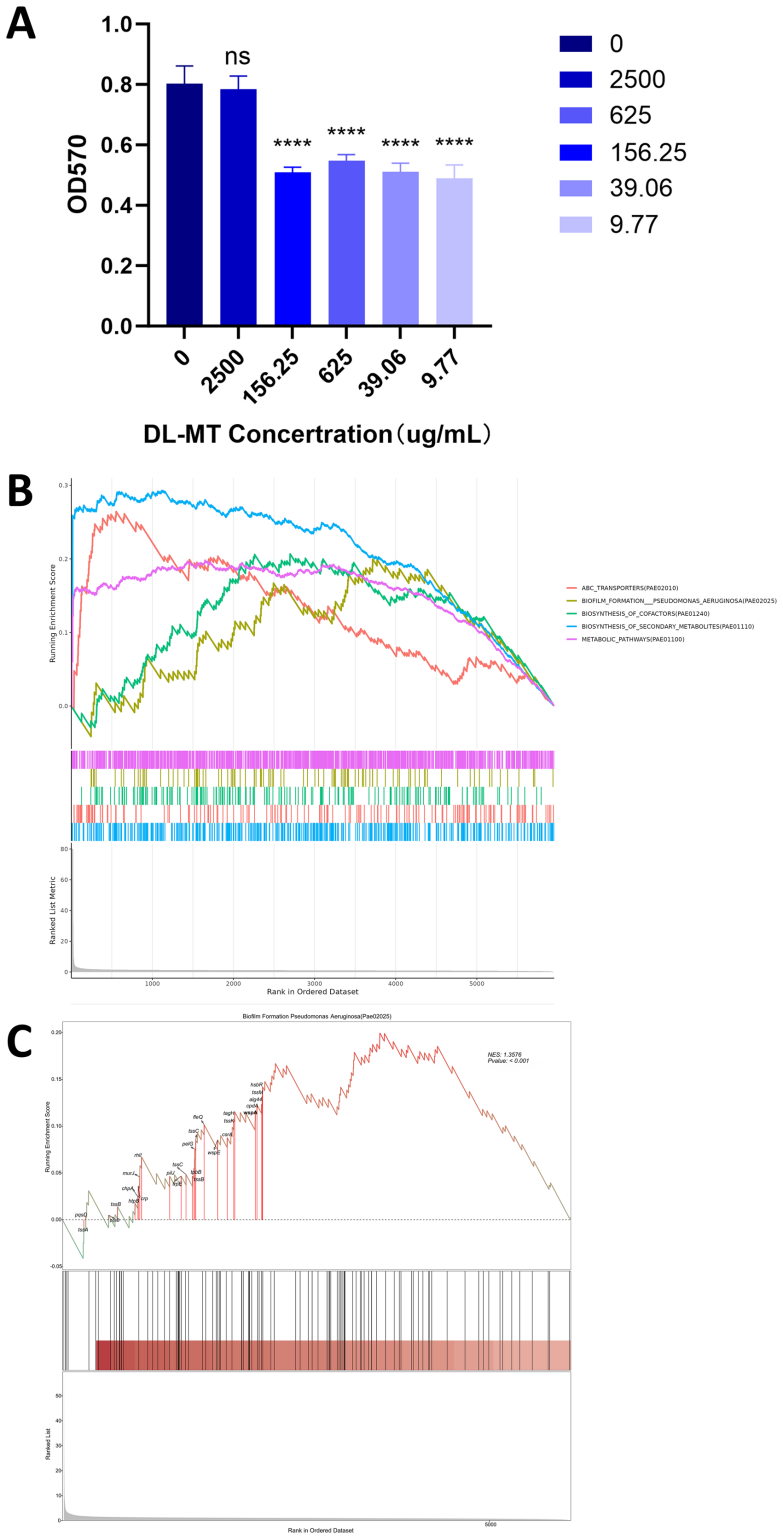
Gene set enrichment analysis results. **(A)**Inhibitory effect of menthol on biofilm formation in *Pseudomonas aeruginosa*. **(B)** Top five pathways most affected by drug treatment. **(C)** Significantly altered genes in the biofilm formation pathway following treatment.

Pathway enrichment analysis of differentially expressed genes revealed significant enrichment of the “Biofilm formation-*Pseudomonas aeruginosa*” pathway (normalized enrichment score > 1, nominal *p* value < 0.05), with key genes such as wspA showing notable expression changes (Figure 10). These findings suggest that the compound-induced transcriptional changes may regulate multiple key aspects of biofilm formation, including the wspA-associated signaling pathway and downstream biofilm-related processes.

## 4 Discussion

The antibiotic resistance and chronic infection capability of *P. aeruginosa* largely depend on its ability to form biofilms (Lebeaux et al., 2014; Maurice et al., 2018). Biofilms limit antibiotic penetration, reduce metabolic activity, and alter local pH, thereby enhancing bacterial tolerance to antimicrobial agents. Biofilm formation represents a highly coordinated microbial response to environmental stimuli, tightly regulated by multiple genes and signaling pathways (Poulsen et al., 2019; Qin et al., 2022). Traditional experimental approaches for identifying key regulatory targets are often time-consuming and limited in efficiency. Recent advances in high-throughput omics and bioinformatics have enabled the rapid and systematic identification of core regulatory genes. Such integrative strategies not only improve the efficiency of target discovery but also provide a foundation for the development of novel interventions against *P. aeruginosa*.

In this study, we identified 73 DEGs associated with biofilm formation based on GEO datasets. GO enrichment analysis indicated that these genes are mainly involved in heme biosynthesis, signal transduction, biofilm formation, and chemotaxis, and are primarily localized to the periplasm, cytoplasm, and plasma membrane. Functionally, they are enriched in electron transfer, oxidoreductase activity, and heme/metal ion binding, suggesting potential roles in energy metabolism, environmental response, and transmembrane signaling (Hartojo and Doyle, 2024; He and Helmann, 2024). KEGG pathway analysis further revealed significant enrichment in nitrogen metabolism, porphyrin metabolism, two-component systems, and biofilm formation pathways, indicating that these DEGs may play key roles in the regulatory network of biofilm formation in *Pseudomonas aeruginosa*.

Among the candidate genes, wspA was enriched in both biofilm formation and two-component system-related pathways and consistently showed strong discriminative performance across multiple machine learning models and independent datasets. It occupies the upstream sensory position of the Wsp–c-di-GMP signaling axis, directly determining downstream biofilm-associated transcriptional outputs. In contrast, htpG, encoding an Hsp90 family chaperone, is mainly involved in protein folding and stress homeostasis and is not directly involved in biofilm-specific signaling pathways, with only indirect effects on biofilm regulation (Buchner, 2010; Hartl et al., 2011). Therefore, wspA more accurately reflects the core regulatory mechanism of biofilm formation.

Importantly, adaptive mutations in wspA can constitutively activate the Wsp pathway, leading to elevated intracellular c-di-GMP levels, enhanced biofilm formation, and rugose small-colony variant phenotypes. This phenotype is closely linked to persistent colonization, environmental adaptation, and chronic infection maintenance, highlighting the critical role of wspA in both biofilm initiation and long-term adaptation (Xu et al., 2022).

Previous studies have confirmed that wspA functions as the primary sensor protein of the Wsp system in *P. aeruginosa*, acting as a MCP-like membrane receptor that detects cell-surface contact and triggers the Wsp phosphorelay cascade (O’Neal et al., 2022). Surface stimulation activates WspE, which phosphorylates WspR and enhances its diguanylate cyclase activity, promoting c-di-GMP accumulation (Huangyutitham et al., 2013). Elevated c-di-GMP inhibits motility and promotes adhesion while inducing Pel-, Psl- and other EPS production, thereby strengthening biofilm matrix formation (Jenal et al., 2017; Zhan et al., 2024). In addition, c-di-GMP signaling is interconnected with quorum sensing and stress response networks, collectively promoting biofilm maturation and persistence (Valentini and Filloux, 2016).

Within this regulatory framework, wspA serves as the upstream sensory hub of the Wsp–c-di-GMP pathway, governing the activation of downstream biofilm-associated transcriptional programs. In contrast, canonical extracellular polysaccharide biosynthesis genes (e.g., pelA, pslA, and algD) and quorum-sensing regulators (e.g., lasR and rhlR) function primarily as downstream effectors whose expression and activity are largely influenced by intracellular c-di-GMP levels. These observations identify wspA as a key link between surface sensing and biofilm regulatory networks, underscoring its pivotal role in biofilm development and its promise as a therapeutic target against biofilm-associated *P. aeruginosa* infections.

Research on wspA inhibitors remains limited. Here, we employed hierarchical virtual screening (HTVS, SP, and XP), followed by clustering analysis, to select candidate compounds. These compounds were further evaluated using molecular dynamics simulations and binding free energy calculations. Most protein–ligand complexes reached equilibrium at approximately 80 ns in molecular dynamics simulations. TMP exhibited instability. CMS is hydrolyzed *in vivo* to generate active colistin, which can efficiently kill *P. aeruginosa* by disrupting the cell membrane and significantly inhibit bacterial growth and biofilm formation (Bergen et al., 2006). CPT-F, a novel cephalosporin, exhibits potent bactericidal activity against *P. aeruginosa*, effectively inhibiting its proliferation and demonstrating stronger effects than conventional cephalosporins (Vidaillac et al., 2009). Given their strong bactericidal effects, the observed biofilm inhibition may, to some extent, result from non-specific antimicrobial pressure rather than targeted biofilm disruption.

To validate the regulatory effect of the virtual screening candidate on biofilm formation and wspA, menthol, a compound with relatively low antibacterial activity, was selected for experimental evaluation. Molecular docking revealed that DL-MT rapidly stabilized within the binding pocket and formed persistent interactions with key wspA residues (ASP227, ASP233, ALA230, and PHE540) through hydrogen bonds, hydrophobic contacts, and water-mediated bridges. Its favorable binding free energy (−28.83 kcal/mol) supports the possibility that DL-menthol may interact with wspA.

Transcriptomic analysis further showed that menthol treatment significantly alters the top five pathways enriched by GSEA, including the biofilm formation pathway of PAO1, with wspA among the differentially expressed genes, suggesting a potential involvement of the Wsp signaling system in the anti-biofilm response to menthol. These findings are consistent with previous reports by Husain et al. (2015), who demonstrated that menthol reduces extracellular polysaccharide production and suppresses elastase and phenazine secretion, leading to looser biofilm structure and decreased adhesion, further supporting the anti-biofilm activity of menthol.

In this study, we employed an integrative approach combining bioinformatics, machine learning, and virtual screening to identify key targets and potential inhibitors involved in *P. aeruginosa* biofilm formation. Our findings provide insights into the molecular mechanisms underlying biofilm regulation and may support the future development of anti-biofilm strategies. Furthermore, this workflow may serve as a useful methodological reference for investigating other biofilm-forming pathogens.

Nevertheless, several limitations remain. Although the anti-biofilm activity of the selected candidate compounds was partially validated *in vitro*, direct experimental validation of the hub gene wspA was not performed. Systematic evaluation at the cellular or animal model level was also lacking, leaving the physiological functions and regulatory effects of this target to be further elucidated. Future studies should combine multi-omics analyses with genetic and experimental verification to systematically map biofilm regulatory networks and guide therapeutic target discovery.

## 5 Conclusion

This study highlights wspA as a key regulator and a candidate target involved in *P. aeruginosa* biofilm formation. Structure-based virtual screening and molecular dynamics simulations identified DL-menthol as a candidate compound with predicted affinity for WspA, while transcriptomic analysis revealed significant effects on biofilm-associated pathways. Together, these findings provide mechanistic insights into *P. aeruginosa* biofilm regulation and offer a foundation for the future development of targeted anti-biofilm strategies.

## Data Availability

The original contributions presented in the study are included in the article/supplementary material, further inquiries can be directed to the corresponding authors.

## Footnotes

1
https://swissmodel.expasy.org/

2
https://seq2fun.dcmb.med.umich.edu//I-TASSER/

3
https://alphafold.ebi.ac.uk/

4
https://saves.mbi.ucla.edu/
